# Structure‐guided envelope trimer design in HIV‐1 vaccine development: a narrative review

**DOI:** 10.1002/jia2.25797

**Published:** 2021-11-21

**Authors:** Ronald Derking, Rogier W. Sanders

**Affiliations:** ^1^ Department of Medical Microbiology Amsterdam Infection & Immunity Institute Amsterdam UMC, AMC University of Amsterdam Amsterdam The Netherlands; ^2^ Department of Microbiology and Immunology Weill Medical College of Cornell University New York New York USA

**Keywords:** broadly neutralizing antibodies, reverse vaccinology 2.0, structure‐based vaccine design

## Abstract

**Introduction:**

The development of a human immunodeficiency virus 1 (HIV‐1) vaccine remains a formidable challenge. An effective vaccine likely requires the induction of broadly neutralizing antibodies (bNAbs), which likely involves the use of native‐like HIV‐1 envelope (Env) trimers at some or all stages of vaccination. Development of such trimers has been very difficult, but much progress has been made in the past decade, starting with the BG505 SOSIP trimer, elucidation of its atomic structure and implementing subsequent design iterations. This progress facilitated understanding the weaknesses of the Env trimer, fuelled structure‐guided HIV‐1 vaccine design and assisted in the development of new vaccine designs. This review summarizes the relevant literature focusing on studies using structural biology to reveal and define HIV‐1 Env sites of vulnerability; to improve Env trimers, by creating more stable versions; understanding antibody responses in preclinical vaccination studies at the atomic level; understanding the glycan shield; and to improve “on‐target” antibody responses versus “off‐target” responses.

**Methods:**

The authors conducted a narrative review of recently published articles that made a major contribution to HIV‐1 structural biology and vaccine design efforts between the years 2000 and 2021.

**Discussion:**

The field of structural biology is evolving at an unprecedented pace, where cryo‐electron microscopy (cryo‐EM) and X‐ray crystallography provide complementary information. Resolving protein structures is necessary for defining which Env surfaces are accessible for the immune system and can be targeted by neutralizing antibodies. Recently developed techniques, such as electron microscopy‐based polyclonal epitope mapping (EMPEM) are revolutionizing the way we are analysing immune responses and shed light on the immunodominant targets on new vaccine immunogens. Such information accelerates iterative vaccine design; for example, by reducing undesirable off‐target responses, while improving immunogens to drive the more desirable on‐target responses.

**Conclusions:**

Resolving high‐resolution structures of the HIV‐1 Env trimer was instrumental in understanding and improving recombinant HIV‐1 Env trimers that mimic the structure of viral HIV‐1 Env spikes. Newly emerging techniques in structural biology are aiding vaccine design efforts and improving immunogens. The role of structural biology in HIV‐1 vaccine design has indeed become very prominent and is unlikely to diminish any time soon.

## 1 INTRODUCTION

After more than 30 years of extensive research, an effective human immunodeficiency virus 1 (HIV‐1) vaccine is still not available. The major challenge for an HIV‐1 vaccine is the enormous viral diversity, and an effective HIV‐1 vaccine should induce an immune response that could cope with this diversity [1, 2, 3, 4]. The only relevant target for vaccine design efforts is the HIV‐1 envelope glycoprotein (Env) trimer, the protein that initiates viral entry and the only target for broadly neutralizing antibodies (bNAbs) [5, 6].

Env is a trimeric protein complex composed of three gp120 and three gp41 subunits that are held together by weak non‐covalent interactions [7, 8]. Env is synthesized as a gp160 polyprotein precursor, which is posttranslationally cleaved in its gp120 and gp41 subunits. During synthesis, the protein is decorated by *N*‐linked glycans that comprise approximately 50% of its total mass [9, 10, 11]. The *N*‐linked glycans play various essential roles in the viral life cycle, for example, in protein folding, infectivity by binding to lectin receptors on immune cells and immune evasion [12, 13, 14, 15, 16, 17].

Approximately 20% to 30% of people living with HIV (PLHIV) develop bNAbs albeit usually after several years of infection [18]. In recent years, many bNAbs have been isolated and some have remarkable neutralization breadth [19]. Passive immunization studies in rhesus macaques have shown that bNAbs can protect against simian/human immunodeficiency virus (SHIV) infection [20, 21, 22]. The protective efficacy of the VRC01 bNAb was tested in two recent randomized clinical trials [23]. The outcome of the trials was that VRC01 was not able to protect against HIV‐1 acquisition overall, but did result in a sieving of VRC01‐resistant viruses, suggesting that VRC01 was able to prevent transmission of VRC01‐sensitive viruses. The implication is that one bNAb specificity will most likely not be enough to induce broad protection, and an effective HIV‐1 vaccine will need to induce multiple neutralizing specificities.

Env protein structures are playing an increasingly important role in vaccine design efforts. Resolving protein structures at the atomic level is necessary to define the surfaces that are accessible for the immune system, to determine sites that are targeted by monoclonal antibodies (MAbs), to design immunogens that mimic the proteins found on viral surfaces and to stabilize the desirable conformations [24]. This approach follows from “reverse vaccinology” in which complete genome sequencing of a pathogen is used to select for surface‐expressed proteins to be used in a vaccine [25]. The term “reverse vaccinology” was repurposed in 2002 to describe the utilization of antibodies to select or design antigens with the appropriate binding properties [26]. A next iteration, “reverse vaccinology 2.0” involves the use of antibodies and high‐resolution structures for the design of vaccine antigens [27].

While these concepts are widely applied in the HIV‐1 vaccine design field, such structure‐based vaccine design approaches also accelerated the development of novel vaccines against other pathogens. An example includes respiratory syncytial virus (RSV), for which vaccine development was accelerated when the crystal structure of the pre‐fusion F glycoprotein was resolved, using the D25 neutralizing antibody (NAb) specific for the pre‐fusion form [28, 29]. Similar to HIV‐1 Env and other class I viral fusion proteins, the RSV F glycoprotein is very unstable and transitions very easily to its post‐fusion conformation. The apex targeting D25 MAb stabilized the F glycoprotein in its pre‐fusion conformation, resulting in the high‐resolution pre‐fusion structure of the protein. This structure allowed for the development of a recombinant pre‐fusion‐stabilized F glycoprotein [24, 28]. As structure‐guided design is now an integral part of HIV‐1 vaccine development, we review here the role of structural biology in defining the sites of vulnerability of HIV‐1 Env, and how new vaccine candidates are designed in an iterative manner by using the structural knowledge of immunogens and the antibodies they induce in (pre)clinical immunization experiments. Our focus is on native‐like Env trimers, but we will treat other approaches where relevant as well.

## 2 METHODS

We conducted a narrative review of the relevant literature focusing on studies that use structural biology to reveal and define HIV‐1 Env sites of vulnerability. This information was used to improve soluble Env trimers by several strategies: (a) by creating more stable versions; (b) to understand antibody responses in preclinical vaccination studies at the atomic level; (c) to understand the glycan shield; and (d) to improve “on‐target” antibody responses versus “off‐target” responses. Peer‐reviewed papers and grey literature that reported on the HIV‐1 Env structure, structural improvement of the Env trimer, Env glycosylation and clinical studies were identified between the years 2000 and 19 April 2021. We searched through grey literature and keyword searches in PubMed for the following terms: reverse vaccinology, reverse vaccinology 2.0, structure‐based vaccine design, HIV‐1 vaccine design, HIV‐1 Env structures, HIV‐1 Env structure‐based vaccine design, HIV‐1 broadly neutralizing antibodies, HIV‐1 Env glycosylation. We also identified studies in the articles’ reference lists.

## 3 DISCUSSION

### 3.1 Structure‐based definition of the Env trimer

“Reverse vaccinology” involves the recombinant generation of the NAb‐relevant surface proteins. However, creating stable mimics of the native Env trimers was not achieved until 2013 (Table 1). Since then, much progress has been made in developing faithful mimics of the HIV‐1 Env spike. A major initial step involved the design of the BG505 SOSIP.664 prototype native‐like Env trimer, based on the clade A transmitter/founder virus BG505 [30]. Several modifications were introduced to stabilize the trimer: a disulphide bond (“SOS”) between residues 501 in the gp120 subunit and 605 in the gp41 subunit; an Isoleucine‐to‐Proline (“IP”) substitution at position 559 in the first heptad repeat (HR1) of gp41 that prevents a helix from forming, thereby locking the protein in the pre‐fusion state; in improved furin cleavage site to facilitate complete precursor cleavage between gp120 and gp41; and the removal of the membrane‐proximal, transmembrane and cytoplasmic tail domains [31]. The BG505 SOSIP.664 trimer closely resembled the native viral Env in both structure and antigenicity [31, 32, 33, 34, 35]. It also allowed obtaining the first medium‐resolution structures of an Env trimer by both cryo‐electron microscopy (cryo‐EM) and X‐ray crystallography techniques. The structures revealed that the gp120 subunits have a globular conformation, which are assembled into a trimer through key interactions in the V1V2 and V3 domains. The gp41 domain forms a pedestal with the gp120 subunits docked on it, combining into a mushroom‐like shape for the functional trimer [36, 37, 38]. The high‐resolution cryo‐EM structure of the JRFL native viral Env also confirmed that the BG505 SOSIP.664 trimer closely mimics viral Env [39] (Table 1). The structure and conformational flexibility of SOSIP trimers were also studied using hydrogen‐deuterium exchange (HDX) and double electron‐electron resonance (DEER) spectroscopy, revealing that these regions are more conformationally flexible than others [40, 41].

**Table 1:** Key developments in structure‐guided HIV‐1 vaccine designs in the past decade

**Year**	**Development**	**Reference**
2013	First structure of a native‐like trimer in complex with PG9	Julien *et al*., 2013 [44]
2013	Development of the BG505 SOSIP.664 trimer	Sanders *et al*., 2013 [31]
2013	First cryo‐EM structure of a native‐like Env trimer	Lyumkis *et al*., 2013 [36]
2013	First crystal structure of a native‐like Env trimer	Julien *et al*., 2013 [37]
2014	First structure of the complete pre‐fusion conformation gp41	Pancera *et al*., 2014 [38]
2014	First dynamics of SOSIP trimers using hydrogen‐deuterium exchange analysis	Guttman *et al*., 2014 [40]
2016	First cryo‐EM structure of a native HIV‐1 viral envelope	Lee *et al*., 2016 [39]
2016	Development of the eOD‐GT8 germline‐targeting inmmunogen	Jardine *et al*., 2016 [118]
2017	Development of the germline‐targeting BG505 SOSIP GT1 trimer	Medina‐Ramirez *et al*., 2017 [125]
2018	Evaluation of site‐specific glycosylation on virion‐derived Envs	Struwe *et al*., 2018; Cao *et al*., 2018 [95, 96]
2018	Analysis of conformational dynamics native‐like Env trimers using DEER spectroscopy	Stadtmueller *et al*., 2018 [41]
2018	First in‐human phase I clinical trial started with the eOD‐GT8 60mer vaccine candidate	Clinicaltrials.gov [136]
2018	First in‐human phase I clinical trial started with a native‐like Env trimer	Clinicaltrials.gov [137]
2018	Development of electron microscopy‐based polyclonal epitope wrapping (EMPEM)	Bianchi *et al*., 2018 [112]
2020	First in‐human phase I clinical trial started with a germline‐targeting native‐like Env trimer	C1inicaltrials.gov [124]

### 3.2 Structure‐based definition of targets of vulnerability on the Env trimer

**Figure 1:**
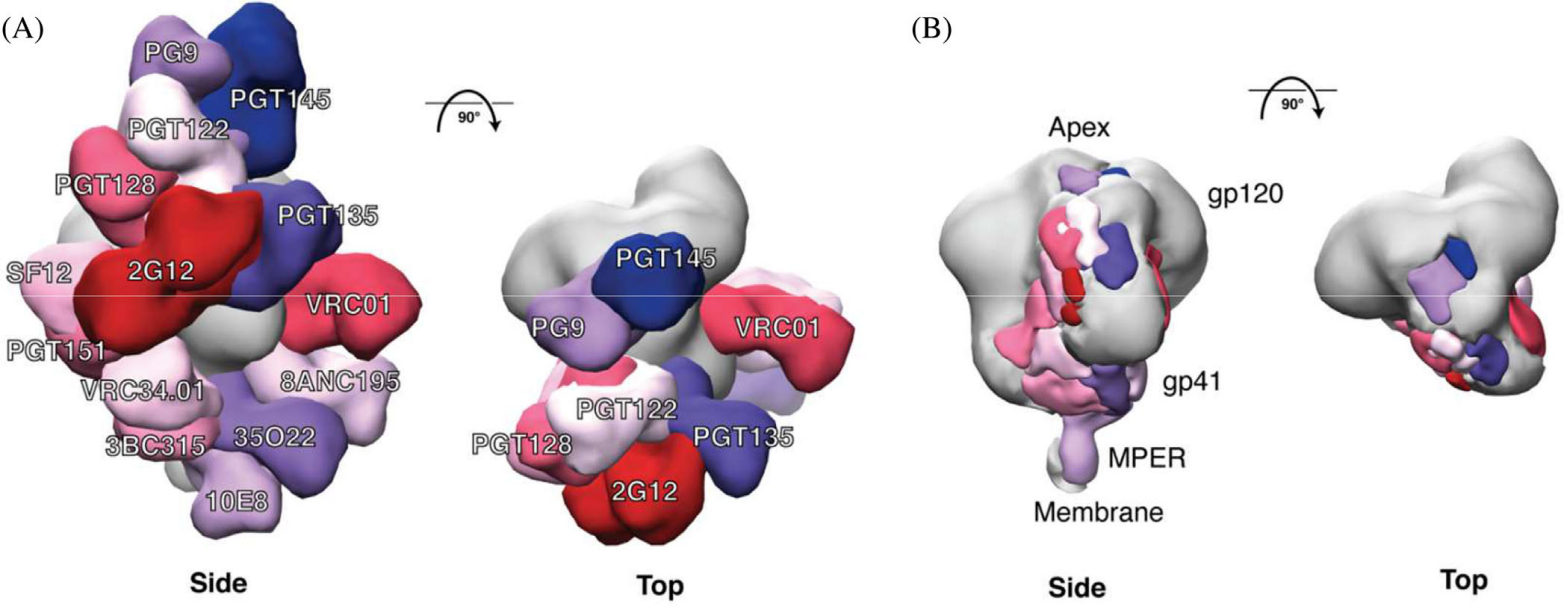
bNAb epitopes mapped onto the three‐dimensional structure of the BG505 SOSIP.664 trimer. **(a)** Side and top views of the bNAbs labelled in different colours that are modelled onto an EM density map of the BG505 SOSIP.664 trimer (coloured in grey). The figure includes bNAbs recognizing eight well‐defined sites of vulnerability: PG9 and PGT145 (V2apex), PGT122 and PGT128 (N332‐glycan); PGT135 and 2G12 (OD‐glycan) both involve the N332 glycan; VRC01 (CD4bs); SF12 (silent face); PGT151, 8ANC195 and 35O22 (gp120‐gp41 interface); VRC34.01 (fusion peptide); 3BC315 (gp41); and 10E8 (MPER). Only one copy of each epitope per trimer is shown for clarity. Thus, the model does not indicate the stoichiometry of bNAb binding, only the location of the epitope. **(b)** Side and top views of the bNAb footprints displayed in (a). This figure is an updated version of fig. 1 from de Taeye *et al*., 2016 [53] (we thank Gabe Ozorowski for preparing it).

A plethora of bNAbs have been isolated from PLHIV that provide templates for vaccine design according to the approaches defined by “reverse vaccinology 2.0”. While structures of bNAbs in complex with gp120 were solved before and generated important structural information on some bNAb epitopes, the availability of the SOSIP trimer allowed many more bNAb epitopes to be determined structurally including epitopes requiring quaternary structures such as those located at the trimer apex and the gp120/gp41 interface [42, 43]. While at very low resolution, the first structure to be described was that of the bNAb PG9 in complex with the BG505 SOSIP.664 trimer [44]. The development of the BG505 SOSIP.664 trimer also helped in revealing many sites of vulnerability on Env [45, 46]. These bNAbs target eight well‐defined clusters comprising of (1) the V1V2 quaternary structure‐dependent epitope cluster, involving the N156 and N160 glycans and electropositive residues in the C‐strand of the V2 [47]. Inducing neutralizing breadth to this epitope can be achieved by a limited amount of mutations, making this an attractive vaccine target [48, 49]. The bNAbs targeting this site possess a very long CDRH3, which is required to navigate through the glycan shield. (2) The N332 centred supersite of vulnerability, which can be subdivided into a V3‐glycan epitope cluster and an outer domain (OD) cluster, but both involve the N332 glycan [46]. This site is being explored in immunogen design efforts discussed below. (3) The CD4 binding site (CD4bs) is targeted by the most potent bNAbs discovered and a main feature is that bNAbs targeting this site need to accommodate the N276 glycan [50]. Many bNAbs to this site, those belonging to the VRC01‐class, are derived from VH1‐2 and have a light chain with a short five amino acid CDRL3. (4) The highly glycosylated “silent” face on Env is a relatively newly discovered epitope that comprises of the glycans N262, N295 and N448. Only a few bNAbs against this site have been isolated, but some of them show remarkable breadth, making the silent face an additional target for vaccine design efforts [51, 52]. (5) The gp120‐gp41 interface comprises of protein domains and glycans of both the gp120 and gp41 subunits and recognize the pre‐fusion closed state of Env [43]. (6) The Fusion peptide epitope cluster comprises of domains in gp120, gp41 and the N‐terminal part of the fusion peptide (FP) [43]. This epitope cluster overlaps with the previous one. (7) The gp41‐domain is targeted by the 3BC176 and 3BC315 bNAbs and these bNAbs neutralize the virus by increasing the decay of the Env trimer [53]. (8) Finally, the membrane proximal external region (MPER) is a highly conserved, hydrophobic region in gp41, and is targeted by bNAbs that are very broad, but have moderate potency [19]. While the above classification of epitopes is useful, in fact, the Env trimer surface appears to form one contiguous target for bNAbs. When multiple bNAbs are plotted on the trimer surface virtually no uncovered surface remains (Figure 1) [46]. Furthermore, while the extensive glycosylation on Env was long thought to create an immunologically “silent face” and act as a “glycan shield” to protect the underlying Env protein domains against NAbs [6, 54], the definition of bNAb epitopes reveals that the glycan shield itself is, paradoxically, a target for bNAbs. As some of these clusters are capable of inducing very potent bNAbs, they are of particular interest for vaccine design efforts, and we further discuss below sites that are being explored as potential vaccine targets.

### 3.3 Structure‐based immunogen improvement

**Figure 2:**
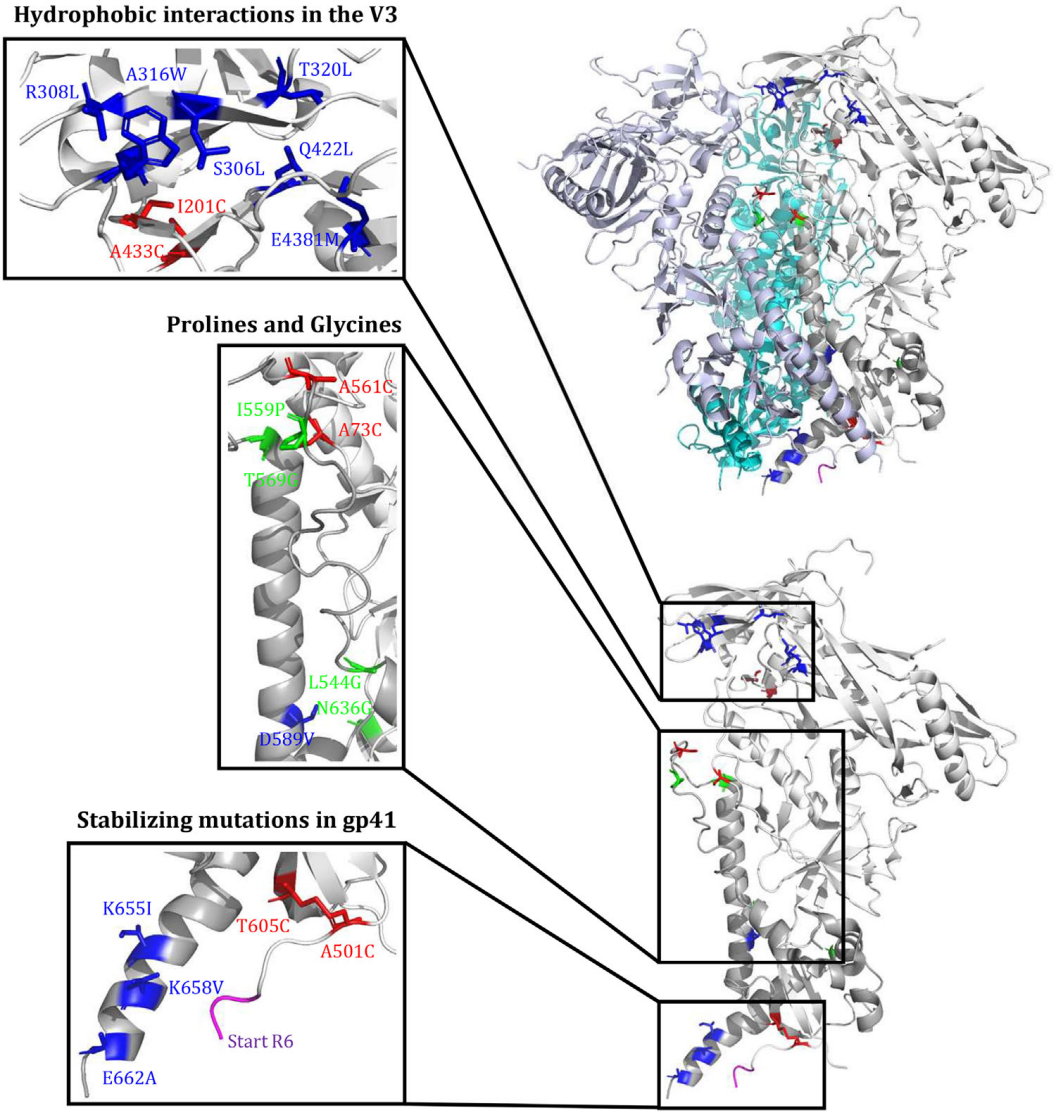
Amino acid substitutions that are routinely used to stabilize soluble native‐like trimers. The amino acid substitutions were modelled on the BG505 SOSIP.664 trimer (see text for details). The gp120 subunit is coloured in white and the gp41 subunit in light gray. In gp120: blue ‐ S306L, R308L, A316W, T320L, E381M and Q422L hydrophobic residues; red ‐ I201C‐A433C and A73C‐A561C disulphide bonds; green ‐ I559P, L544G, T569G and N636G stabilizing mutations; magenta ‐ adjacent residues of the R6 furin cleavage site or flexible linkers. In gp41: red ‐ A501C‐T605C disulphide bond; blue ‐ D589V, K655I, K658V and E662A hydrophobic stabilizing mutations. A more extensive list of stabilizing mutations was reviewed previously [55].

While class I viral fusion proteins are intrinsically unstable, a number of tricks can be applied to stabilize them in their pre‐fusion conformation. We briefly review a few important ones that are now routinely used, but there are other possibilities (Figure 2). For a more extensive review, see Torrents de la Peña *et al*., 2018 [55].

#### 3.3.1 Trimer stabilization using disulphide bonds

The initial step of the SOSIP design involved the introduction of a well‐placed disulphide bond between gp120 and gp41 described over two decades ago [32]. The structures of the BG505 SOSIP.664 trimer sparked the designs of more stable versions of the Env trimer by adding additional disulphide bonds. Such hyperstabilization of native‐like Env trimers can reduce their flexibility and increase their stability, which could be beneficial for promoting interactions of B‐cell receptors (BCRs) with bNAb epitopes. Several studies increased the stability of the Env trimer by introducing disulphide bonds [56, 57]. The DS‐Env trimer, a variant of the BG505 SOSIP.664 trimer, is conformationally fixed by a disulphide bond at positions 201 and 433 that prevents conformational changes associated with CD4 binding, resulting in reduced binding of non‐NAbs targeting the V3 and CD4bs [57]. Torrent de la Pena *et al*., created a hyperstable BG505 SOSIP trimer by introducing several new disulphide bonds, including one between gp120 and gp41 from different protomers covalently linking them, leading to improved stability and antigenicity [58]. A similar approach was used to stabilize the glycoprotein complex (GPC) of Lassa virus (LASV), and involved a disulphide bond to covalently link the GP1 and GP2 subunits [59].

#### 3.3.2 Trimer stabilization using prolines

The second critical component of the original SOSIP design involves a proline substitution in HR1 that prevents conformational changes down the fusion pathway, thereby stabilizing the Env trimers in the pre‐fusion state. Proline substitutions at other positions, as well as glycine substitutions, can have similar stabilizing effects [34, 60, 61]. This method proved to be a universal method for stabilizing class I fusion proteins. Its next use was for the stabilization for RSV F proteins, but applications to LASV GPC [59], Ebola virus GP, and MERS‐CoV and SARS‐CoV Spike (S) proteins swiftly followed [62, 63, 64, 65]. The latter configuration was adopted in SARS‐CoV‐2 vaccine design, and the Pfizer/BioNTech, Moderna, Curevac, Janssen/J&J, and Novavax COVID‐19 vaccines all include the proline stabilization method [66, 67].

#### 3.3.3 Trimer stabilization using hydrophobic residues

While the BG505 SOSIP.664 trimer is a good mimic of the native Env spike, it can still fluctuate between a closed and more open state(s). This flexibility can lead to the exposure of the V3 [68, 69, 70]. Several strategies to further stabilize the trimer involved further reducing flexibility and silencing of the immunodominant V3. Using structure‐guided design, the A316W mutation was introduced to strengthen the hydrophobic interactions at the trimer apex, reducing the propensity of the V3 to “flip out” of its position underneath the V1V2 domain [71]. The introduction of this tryptophan significantly reduced the V3‐directed responses in rabbits and mice [71]. By using structure‐based design and mammalian display, other hydrophobic residues were introduced between the V3 and V1V2‐domains to reduce spontaneous exposure of the V3 by introducing leucine residues at positions 306 and 308 to create hydrophobic interactions with the tryptophan at position 316, by the “so‐called” MD39 mutations, in the trimer core (A204I, T320L, E381M, Q422L), or in gp41 (D589V, K655I, K658V, E662A) [72, 73, 74, 75, 76].

#### 3.3.4 Trimer stabilization by removing the furin cleavage site

The SOSIP platform includes an improved furin cleavage site to allow for complete precursor cleavage, which requires coexpression of the furin protease. This configuration was also applied successfully to LASV GPC. The BG505 SOSIP structures allowed for the design of single chain (SC) or native flexibly linked trimer (NFL) platforms, in which the furin cleavage site is replaced by a flexible linker allowing for the production of native‐like trimers independent of the presence of furin [60, 77, 78, 79]. These flexible linkers have a specific length to allow for the natural association of the Env subunits, and these platforms often contain some or all of the SOSIP modifications to assist in stabilization. Replacement of the cleavage site in combination with proline stabilization has also aided the development of SARS‐CoV‐2 vaccines [66].

### 3.4 Broadening the panel of native‐like trimers

The enormous sequence diversity of HIV‐1 is a key challenge in designing an effective vaccine. The vaccine should induce bNAb responses that can cope with this diversity [1, 2, 3, 4]. For MAbs to be effective against highly mutable pathogens, they have to undergo a mutation process called somatic hypermutation (SHM), to produce high‐affinity MAbs that can tolerate this sequence diversity. Developing immunization strategies that drive this process will be key for an effective HIV‐1 vaccine. *In silico* studies revealed that cocktail and sequential immunization regiments could be good strategies to drive the SHM process [80, 81, 82]. Therefore, increasing the number of native‐like trimers based on different genotypes would be a good starting point. The high‐resolution structures of the BG505 SOSIP.664 trimer helped in the designs of more stable versions of this protein and helped in the identification of residues involved in its inherent stability [31, 36, 37, 38, 58, 71, 79]. The identification of these residues made it possible to successful apply specific stabilizing mutations to other Env sequences. In recent years, many native‐like trimers, based on different platforms, have been developed including ones based on a clade A sequence (92UG037.8), clade B sequences (B41, AMC008, AMC009, AMC011, JRFL), clade C sequences (DU422, ZM197M, CZA97.012, 16055), recombinant B/C sequence (LT5.J4b12C) and consensus sequences [56, 57, 60, 69, 71, 73, 74, 75, 76, 77, 78, 79, 83, 84, 85, 86, 87, 88, 89]. The generation of native‐like trimers from any desired Env sequence was taken a step further by Rutten *et al*., 2018, who devised a universal approach in order to obtain diverse high‐quality pre‐fusion closed Env trimers [76]. A structure‐based screening approach was used to develop a “repair and stabilize” (RnS) method, by removing rare amino acids and optimizing regions critical in the folding process. This approach was applicable to diverse HIV‐1 Env trimers in order to improve expression and stabilization [76]. The procedure was improved by the development of a pipeline using an automated process called ADROITrimer. This process combined the DS‐SOSIP design with RnS to create a large number of pre‐fusion‐stabilized Env trimers [90].

### 3.5 Structure‐based interpretation of the glycan shield

Concurrent with the developments in the Env structural biology field, new mass spectrometry (MS) glycan analysis platforms facilitated the dissection of glycans with unprecedented detail. These techniques, when applied to native‐like recombinant trimers, allowed for a detailed characterization of the Env glycan shield, including the composition of each glycan, the level of occupancy at potential *N*‐linked glycosylation site (PNGS) and how this affects binding of bNAbs [91].

HIV‐1 Env has a particular dense glycan shield and this density imposes steric constrains on early glycan trimming enzymes, leading to the large abundance of underprocessed oligomannose‐type glycans [91]. These underprocessed glycans form two specific “mannose patches” on Env: (a) the intrinsic mannose patch (IMP), which is highly conserved among all HIV‐1 clades; and (b) the trimer‐associated mannose patch (TAMP), which only forms on native‐like Env trimers [92]. Glycan density thus influences glycan processing and epitope presentation. This was corroborated when PNGS surrounding the 2G12 epitope were removed, thereby increasing the processing of these glycans, which impacted 2G12 binding. A similar effect was observed for the N160 glycan, which showed increased processing after the removal of the N156 or N197 sites, affecting the binding of apex targeting bNAbs [93]. These examples underpin the importance of understanding the role of glycan networks and glycan composition on conserving bNAb epitopes for HIV‐1 Env immunogen design. The glycan density was shown to be important for the induction of neutralizing breadth, as during natural HIV‐1 infection viruses that have an intact glycan shield are more prone to induce bNAb responses compared to the ones that have holes in the glycan shield [94]. The BG505 sequence lacks the conserved N241 and N289 PNGS and in the BG505 infant, neutralization breadth was first observed after the introduction of the N241 glycan showing the importance of filling glycan holes [94].

At least equally important from an immunogen design perspective was the quantitative measurement of PGNS occupancy on both viral Envs and recombinant SOSIP trimers, showing that several sites on recombinant trimers are underoccupied [95, 96]. Underoccupancy creates artificial glycan holes that are immunogenic, but induce NAb responses that are unable to neutralize the corresponding virus on which the respective sites are removed [97, 98]. The importance of artificial glycan holes will be discussed below.

The Env glycans and the glycan shield are conformationally dynamic, much more so than the underlying Env protein domains. A recent study described an approach that combined cryo‐EM, computational modelling and MS to visualize the glycan shield structure and dynamics [99]. This approach was able to detect subtle changes in PNGS occupancy, glycan compositions and glycan dynamics that can impact the structure of the glycan shield and epitope accessibility [99]. Further *in silico* studies using molecular dynamics simulations attempted to predict glycan movement for HIV‐1 and SARS‐CoV‐2 [100, 101]. A better understanding and visualization of the dynamics of the glycan shield helps to understand how bNAbs cope with the glycan shield and how we need to consider this in vaccine design efforts.

### 3.6 Structure‐based analysis of Ab responses against native‐like trimers

#### 3.6.1 Mapping Ab responses by traditional serology

The extensive studies on the prototype BG505 SOSIP.664 trimer in rabbit and rhesus macaque immunization studies underlined how the glycan shield ‐ and its holes ‐ shape the NAb response [70]. Serum neutralization experiments with mutant viruses revealed several NAb specificities, but with limited breadth. A major proportion of the autologous Tier‐2 NAb responses was directed to the large glycan hole around positions 241 and 289, which is specific to the BG505 strain [70, 97]. The serum neutralization experiments also identified neutralizing epitopes residing in the V1 and C3/V5 regions [102, 103, 104]. The BG505 SOSIP.664 trimer also induced strong Tier‐1 neutralizing responses targeting a linear epitope in the V3 of gp120. The V3 is not exposed on most primary viruses, and these specificities are therefore unable to neutralize such viruses [70]. Finally, the removal of the transmembrane domain and cytoplasmic tail creates a neo‐epitope at the trimer base, which is immunodominant [105]. Animal vaccination studies using NFL trimers yielded very similar results as BG505 SOSIP trimers, with strain‐specific responses to the N241/N289 glycan hole dominating [106, 107].

#### 3.6.2 Mapping Ab responses by structural analysis of MAbs from immunized animals

Traditional serology studies are sometimes difficult to interpret because the mutations might have allosteric effects. Furthermore, they do not reveal approach angles, atomic contacts and other structural features. MAbs have been isolated from several animal species immunized with the BG505 trimer [97, 102, 108, 109, 110, 111]. EM analysis of these MAbs in complex with the BG505 trimer revealed that many of the strain‐specific NAbs indeed targeted the N241/N289 glycan hole created by the absence of these glycans [97, 102]. Other studies identified sites leading to narrow‐neutralization breadth, in the C3/V4, C3/V5 and the V1 regions [102, 108, 109, 110, 111]. While these specificities are unlikely to be amendable to developing neutralizing breadth, occasionally MAbs are found that are. An example is a BG505 trimer‐induced MAb that targets the gp120/gp41 interface with significant overlap with the epitope of the VRC34 bNAb [98].

#### 3.6.3 Mapping Ab responses by EMPEM

MAb isolation is a laborious process and it always remains unclear how representative a given MAb is for the overall serological response. In addition, it is difficult to appreciate the totality of the polyclonal response based on a limited set of MAbs. Electron microscopy‐based polyclonal epitope mapping (EMPEM) is a recently developed technique that allows for a visual snapshot of the specificities present in serum samples [111, 112] (Table 1). The values of the EMPEM technique in delineating serum responses was revealed as it uncovered previously unidentified epitopes (described below), but also confirmed the known N241/N289 glycan hole and base targeting specificities [112]. The newly discovered antibody specificities included ones that target the cleft of the trimer (COT) between two protomers, and one around the N241/N289 glycan hole with no neutralization activity [112]. In macaques, similar responses were observed, but also additional epitopes were identified targeting the fusion peptide, V1/V3 and an epitope in the gp120/gp41 interface [111]. The base specificities are not new, but EMPEM analysis revealed that the base responses were dominating very early after the first prime [112]. The visualization of the different specificities present in one serum is a major asset to the toolkit to analyse antibody serum responses. When performed in sequential samples, it also allows visualizing the evolving antibody specificities over time, in response to different immunizations.

### 3.7 Structure‐based modification of the glycan shield

#### 3.7.1 Filling holes in the glycan shield

**Figure 3:**
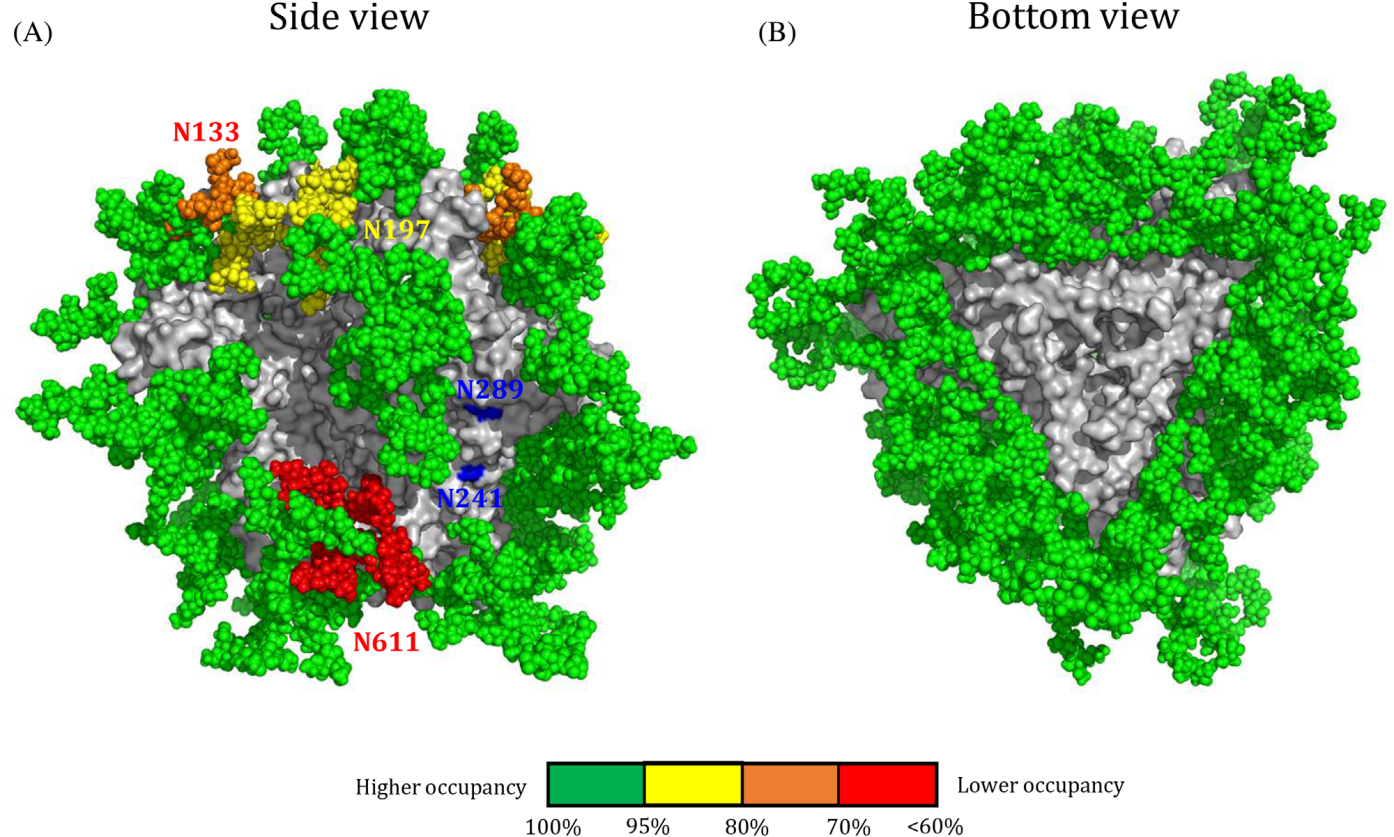
Sources for glycan holes found on stabilized soluble native‐like trimers. **(a)** Side and bottom view of the glycosylated BG505 SOSIP.664 trimer. The glycans were modelled on the crystal structure of BG505 SOSIP.664 (PDB: 5CEZ) using the Glyprot tool from Glycosciences. The missing glycans at positions 241 and 289 on gp120 are indicated in blue. Colour coding reflects the occupancy of each PNGS, similar as described in [114]. Green, full occupancy (>95%); yellow, 80% to 95%; orange, 70% to 80%; red, >60% occupancy. Lower occupancy of a PNGS results in an artificial glycan hole that can be immunogenic. **(b)** Bottom view of the glycosylated BG505 SOSIP.664 trimer. The removal of the trimer from the membrane creates a large immunodominant hole.

While glycans play important roles in bNAb responses, holes in the glycan shield caused by the absence of PNGS that are present in most primary virus isolates appear to dominate the antibody response after vaccination with Env trimer immunogens. These glycan holes induce NAb responses of narrow breadth [70, 97, 113]. Filling glycan holes could therefore be a strategy to focus the immune response to more desirable targets. This strategy has been successfully applied to BG505, where the absence of the highly conserved PNGS at positions 241 and 289 in the BG505 sequence creates a large immunodominant hole that induces the majority of the strain‐specific NAbs (Figure 3) [70, 97]. As the NAb responses to this hole are “dead‐end” specificities that cannot be broadened, filling this hole may be a good strategy, and it can easily be achieved by restoring the missing PNGS [102, 104, 113].

#### 3.7.2 Filling artificial holes in the glycan shield

Glycan holes can also exist by underoccupancy of PNGS, creating artificial glycan holes that can distract the immune system from more desirable targets. Several PNGS are underoccupied on BG505 SOSIP trimers, while they are fully occupied on the parental virus (Figure 3). The fact that holes created by PNGS underoccupancy are immunogenic was revealed by isolation of MAbs from BG505 SOSIP.664 immunized rhesus macaques that target the N611 site. These MAbs can only neutralize when the N611 glycan is removed, indicating that the site is fully occupied on the virus [70, 97, 98]. High‐resolution structures of Env in complex with the N611 targeting non‐NAb RM20E1 revealed that the N611 glycan is indeed underoccupied to an extend of approximately 60% [98, 114]. A sequon engineering strategy increased the occupancy of several PNGS on the BG505 SOSIP trimer, as well on several other Env isolates, thereby eliminating artificial glycan holes [114]. The strategy exploited observations on model proteins from the mid‐1990s that N‐X‐T sequons have a two to three‐fold higher propensity to become occupied compared to N‐X‐S sequons [115]. The replacement of N‐X‐S sequons by N‐X‐T sequons enhanced PNGS occupancy overall, although some site‐specific adjustments needed to be made [114]. Increasing PNGS occupancy should reduce off‐target responses and steer the immune response to more desirable targets.

#### 3.7.3 Covering the trimer base using glycans

MAb isolation and in particular EMPEM analyses revealed that the trimer base is highly immunodominant after immunization with soluble Env trimers. The truncation of the Env trimer from the membrane, in effect creates a large glycan hole that is highly immunogenic but only induces non‐NAbs because it is absent from virus‐associated Env (Figure 3). As these responses might also be distracting from more desirable NAb responses, covering this large hole might therefore be necessary. This might not be very straightforward. Kulp *et al*. (2017) addressed this by redesigning a BG505 trimer by swapping the orientation of gp120 and gp41 subunits and placing a linker between the two subunits [73, 75]. This linker allowed for the introduction of PNGS to shield the glycan base, resulting in reduced binding of base‐targeting non‐NAbs [75]. Additional efforts to close this hole on Env‐based immunogens are underway and include the presentation of Env on nanoparticles [116, 117]. In a similar approach, glycans were used to mask the “backside” of the eOD‐GT8 immunogen (see below: CD4 binding site‐focused approaches) resulting in the MUT16 immunogen [118, 119].

#### 3.7.4 Introducing new holes in the glycan shield

Filling the N241/N289 glycan hole on BG505 SOSIP trimers reduces the responses directed to this specific site. Opening holes elsewhere on the trimer, by knocking‐out a PNGS can redirect the immune response to these newly formed holes, providing important proof of concept that this strategy might be valuable [104]. Such strategies are being explored to focus antibody responses to the CD4bs by removing glycans that restrict access to this bNAb epitope cluster [120, 121, 122]. In anecdotal cases, this has led to the elicitation of cross‐neutralizing antibody responses directed to the CD4bs [120, 123, 124]. Removing glycans might be particularly relevant for germline‐targeting immunogen approaches, that is, approaches based on targeting and activating bNAb precursor B cells with specific genetic signatures [118, 119, 125]. One component of germline‐targeting design often involves enhancing accessibility of the site of interest, while another component involves the structure‐based design of modifications to enhance the interaction with desirable naïve B cells.

### 3.8 Role of native‐like trimer immunogens in driving bNAb responses

In the above sections, we described the development and fine‐tuning of native‐like trimers, what type of antibody specificities they induce and how they can be further modified. However, they are unlikely to induce bNAb responses by themselves, but they form a platform for further, epitope‐specific, immune focusing and/or they provide the final “polishing” steps to finish off a sequential vaccination regimen to mature antibody lineages to become broadly neutralizing (see also Williams *et al*. [126]). We review three prominent epitope‐focused approaches that involve native‐like trimers at some stage in the regimen. The structures of bNAbs in complex with the HIV‐1 Env trimer and the delineation of the sites of vulnerability on Env are the basis of immunogen designs that focuses on specific epitopes. Epitope‐focused vaccines can be “germline‐agnostic” embracing antibodies derived from different genetic germline signatures, or they can target specific germline classes [43].

#### 3.8.1 Fusion peptide‐focused vaccine approach

While it has long been thought that the FP was hidden inside the interior of the pre‐fusion Env trimers and was poorly immunogenic, the highly conserved FP became a prominent target following the discovery of the FP targeting bNAbs VRC34 and ACS202 [127, 128, 129]. Xu *et al*. investigated the ability of a simple FP peptide to prime FP‐directed Ab responses, choosing the eight *N*‐terminal residues of the FP as these where shown to be contacted by VRC34 [127, 129]. Structure‐based design was used to engineer FP‐containing immunogens that comprised epitope scaffolds and native‐like Env trimers. The FP scaffolds were created by conjugating the eight *N*‐terminal residues, as well as the N88 glycan that contributes to the VRC34 and ACS202 epitopes to keyhole limpet haemocyanin (KLH) as a carrier [129]. An immunization scheme evaluated in nonhuman primates with five FP‐KLH primes followed by three trimer boosts, led to neutralizing breadth in a few animals, showing that FP epitope‐focused vaccine design merits further study [129].

#### 3.8.2 N332 supersite‐focused approaches

The relatively conserved V3‐glycan “supersite” is also an interesting target, as very potent bNAbs are induced against this epitope. Furthermore, the majority of PLHIV produce glycan‐dependent bNAbs to this epitope [130]. The PGT121‐class bNAbs, targeting the N332 supersite in the V3, belong to the most potent and well‐characterized bNAbs described today. Steichen *et al*. designed a gl‐targeting gp120 molecule that specifically binds PGT121 gl‐reverted MAbs, by using mammalian display‐directed evolution [73]. These mutations were then transferred to a stabilized BG505 trimer, resulting in a trimer termed 11MUT_B_. Multivalent display of these trimers on liposomes enhanced the activation of B cells that express gl‐PGT121 as their BCR, compared to the soluble trimers, and elicited MAbs with neutralization breadth in a KI‐mouse model [73, 131]. The RC1 trimer, based on 11MUT_B_, lacks the N133 and N137 glycans but also the N156 glycan as it was hypothesized that this would further improve binding of PGT121‐class precursors [132]. In a further iteration, termed RC1‐4fill, PNGS at positions 230, 241, 289 and 344 were introduced to fill glycan holes (see above) and focus the immune response further to the N332 supersite in the V3. The designs were able to activate and expand B cells in mice, rabbits and macaques that resemble the human V3‐glycan “supersite” targeting germline‐MAbs [132].

#### 3.8.3 CD4 binding site‐focused approaches

The CD4bs is an interesting site for epitope‐focused vaccine design because some of the most potent bNAbs target this functionally conserved site. The VRC01‐class of CD4bs bNAbs have received much attention in this regard because VRC01‐class bNAbs have been identified in multiple PLHIV, and they have distinct genetic signatures that include a VH1‐2‐derived heavy chain and a light chain with a short five amino acid CDRL3. Designing immunogens that could activate gl‐VRC01‐class B‐cell precursors *in vivo* might be key in developing an effective vaccine that can induce bNAbs targeting the CD4bs. Native‐like trimers, such as SOSIP trimers, do not readily induce CD4bs‐directed MAbs very easily nor do they interact with gl‐VRC01‐class MAbs. Reasons are steric constraints because of the location of the CD4bs in the cleft between two protomers, steric hindrance by the N276 glycan and specific atomic contacts with the gl‐MAbs [133, 134]. The development of the VRC01 lineage was most likely initiated by viruses that lacked the N276 glycan [135]. Several immunogens have been designed to activate precursor VRC01‐class B cells. These immunogens include an engineered outer domain (eOD‐GT8) and the BG505 SOSIP GT1 trimer [118, 125, 136]. An updated version of this trimer (BG505 SOSIP GT1.1) entered a phase I clinical trial, as did eOD‐GT8 that showed very encouraging results [124, 137, 138]. In addition to germline targeting approaches for the CD4bs, other germline agnostic approaches involved the deletion of glycans to approach accessibility of the CD4bs [120, 121, 122].

## 4 CONCLUSIONS

The development of the native‐like BG505 SOSIP.664 trimer and its first high‐resolution structure fueled the field of Env structure‐based vaccine design. The Env trimer structures allowed for the identification of modifications to improve the stability, antigenicity and yield of native‐like trimer vaccine candidates, but also allowed for the development of native‐like trimers based on different isolates and clades. Native‐like SOSIP trimers have now entered phase I clinical trials[124, 139, 140, 141, 142, 143, 144] (Table 1; Kim *et al*. [145]), and the outcome of these trials will inform whether native‐like Env vaccine candidates will induce autologous Tier‐2 NAb responses in humans. High‐resolution structures of bNAbs and their germline counterparts in complex with native‐like HIV‐1 Env trimers aided in the identification of their epitopes. New emerging techniques, such as EMPEM, allowing for an increasing understanding of what immunogens induce, will further guide iterative vaccine design efforts with increasing speed. This information will help in reducing undesirable off‐target responses and improve driving on‐target responses towards the development of bNAbs. Finally, rationally designed nanoparticle platforms, not reviewed here, facilitate the multivalent presentation of Env trimers, thereby increasing B‐cell activation and enhancing the magnitude of the antibody response.

## COMPETING INTERESTS

The International AIDS Vaccine Initiative (IAVI) has previously filed a patent relating to the BG505 SOSIP.664 trimer: U.S. provisional application 61/772,739, entitled “HIV‐1 Envelope Glycoprotein”, with R.W.S. among the co‐inventors.

## AUTHORS’ CONTRIBUTIONS

RD: Writing, original draft preparation and figure preparation. RWS: Writing, review and editing.

## FUNDING

Work by the authors in this area is supported by the U.S. National Institutes of Health under grant P0 AI110657; by the Bill and Melinda Gates Foundation through the Collaboration for AIDS Vaccine Discovery (CAVD), grants OPP1132237 and INV‐002022; by the European Union's Horizon 2020 research and innovation programme under grant agreement No. 681137; and by a Vici grant from the Netherlands Organization for Scientific Research (NWO).
